# Automatically Selecting a Suitable Integration Scheme for Systems of Differential Equations in Neuron Models

**DOI:** 10.3389/fninf.2018.00050

**Published:** 2018-10-08

**Authors:** Inga Blundell, Dimitri Plotnikov, Jochen M. Eppler, Abigail Morrison

**Affiliations:** ^1^Institute of Neuroscience and Medicine (INM-6), Institute for Advanced Simulation (IAS-6), Jülich Aachen Research Alliance BRAIN Institute I, Forschungszentrum Jülich, Jülich, Germany; ^2^Simulation Lab Neuroscience, Institute for Advanced Simulation, Jülich Aachen Research Alliance, Jülich Supercomputing Centre (JSC), Forschungszentrum Jülich, Jülich, Germany; ^3^Chair of Software Engineering, Jülich Aachen Research Alliance, RWTH Aachen University, Aachen, Germany; ^4^Faculty of Psychology, Institute of Cognitive Neuroscience, Ruhr-University Bochum, Bochum, Germany

**Keywords:** integrate-and-fire neuron, model dynamics, numerics, integration schemes, ODE, symbolic analysis

## Abstract

On the level of the spiking activity, the integrate-and-fire neuron is one of the most commonly used descriptions of neural activity. A multitude of variants has been proposed to cope with the huge diversity of behaviors observed in biological nerve cells. The main appeal of this class of model is that it can be defined in terms of a hybrid model, where a set of mathematical equations describes the sub-threshold dynamics of the membrane potential and the generation of action potentials is often only added algorithmically without the shape of spikes being part of the equations. In contrast to more detailed biophysical models, this simple description of neuron models allows the routine simulation of large biological neuronal networks on standard hardware widely available in most laboratories these days. The time evolution of the relevant state variables is usually defined by a small set of ordinary differential equations (ODEs). A small number of evolution schemes for the corresponding systems of ODEs are commonly used for many neuron models, and form the basis of the neuron model implementations built into commonly used simulators like Brian, NEST and NEURON. However, an often neglected problem is that the implemented evolution schemes are only rarely selected through a structured process based on numerical criteria. This practice cannot guarantee accurate and stable solutions for the equations and the actual quality of the solution depends largely on the parametrization of the model. In this article, we give an overview of typical equations and state descriptions for the dynamics of the relevant variables in integrate-and-fire models. We then describe a formal mathematical process to automate the design or selection of a suitable evolution scheme for this large class of models. Finally, we present the reference implementation of our symbolic analysis toolbox for ODEs that can guide modelers during the implementation of custom neuron models.

## 1. Introduction

In common with all body cells, nerve cells (*neurons*) are delimited by a bi-lipid layer (the *cell membrane*) which is largely impermeable for ions and bigger molecules. Active ion pumps and passive channels embedded into the membrane allow the selective passage of certain ions. Through these transporter molecules, neurons maintain a gradient of different ion types across the membrane, which leads to the *membrane potential* (Kandel et al., 2013).

In the absence of input, the membrane potential fluctuates around the *resting potential* *E*_L_ (typically at around −70 mV). Excitatory input depolarizes the membrane, driving the membrane potential closer to zero, while inhibitory input hyperpolarizes the neuron, driving the membrane potential away from zero. If the membrane potential crosses the *spiking threshold* θ (typically at around −55 mV), the neuron fires an action potential (*spike*), which is transmitted to all downstream (*postsynaptic*) neurons, where it in turn elicits excursions of their membrane potentials.

The basic integrate-and-fire model describes the dynamics of the membrane potential in the following way: the time evolution of the membrane potential *V* is governed by a differential equation of the type

(1) $$\begin{array}{l} \frac{d}{dt}V\left(t\right)=R\left(V\left(t\right),\cdot \right) \end{array}$$

where *R* can be a function of other variables alongside *V*, whose time evolution is described by another ordinary differential equation which can again contain the membrane potential:

$$\begin{array}{l} \frac{d}{dt}\text{X}=\frac{d}{dt}\left(\begin{array}{c} V\\ x_{1}\\ \vdots \\ x_{n} \end{array}\right)\left(t\right)=\left(\begin{array}{c} R_{0}\left(\text{X}\right)\\ R_{1}\left(\text{X}\right)\\ \vdots \\ R_{n}\left(\text{X}\right) \end{array}\right) \end{array}$$

Once the membrane potential reaches its threshold θ, a spike is fired and the membrane potential is set back to *E*_L_ for a certain amount of time called the *refractory period*. After this time the evolution of equation 1 starts again. An important simplification in most models compared to biology is that the exact course of the membrane potential during the spike is either completely neglected or only considered partially. Threshold detection is typically added algorithmically on top of the sub-threshold dynamics.

The two most common variants of this type of model are the *current-based* and the *conductance-based* integrate-and-fire model. For the current-based model we have the following general form of the equation:

(2) $$\begin{array}{l} \frac{d}{dt}V\left(t\right)=\frac{1}{\tau }\left(E_{\text{L}}-V\left(t\right)\right)\\ +\frac{1}{C}I\left(t\right)+F\left(V\left(t\right)\right). \end{array}$$

Here *C* is the *membrane capacitance*, τ the *membrane time constant* and *I* the *input current* to the neuron. If we assume that spikes are constrained to a fixed temporal grid, *I*(*t*) represents the sum of the currents elicited by all incoming spikes at all grid points for times smaller than *t*, plus a piece-wise constant function *I*_ext_ that models additional external input. *F*, in contrast to the first part of the right-hand-side of equation 2, is some non-linear function of *V* that may also be zero.

For the conductance-based integrate-and-fire model we have:

(3) $$\begin{array}{l} \frac{d}{dt}V\left(t\right)=\frac{1}{\tau }\left(E_{\text{L}}-V\left(t\right)\right)\\ +\frac{1}{C}G\left(t\right)\left(V\left(t\right)-E\right)+F\left(V\left(t\right)\right). \end{array}$$

*G* has the same form as *I* but models a conductance rather than a current. *E* is the *reversal potential* at which there is no net flow of ions from one side of the membrane to the other (for details see Kandel et al., 2013). Equation 3 will usually contain several summands $\frac{1}{C}G_{i}\left(t\right)\left(V\left(t\right)-E_{i}\right)$ for differing *G*_*i*_ and corresponding *E*_*i*_, e.g., for inhibitory and excitatory synaptic conductance. For simplicity we assume only one summand. The differential equations for both the current- and conductance-based models are linear when *F* ≡ 0. For the current-based model this means that equation 2 is a linear *constant coefficient* differential equation.

An example of a neuron model described by a system of differential equations, where *F* ≢ 0 is the *adaptive exponential integrate-and-fire model*:

$$\begin{array}{l} \frac{d}{dt}V\left(t\right)=\frac{1}{\tau }\left(E_{\text{L}}-V\left(t\right)\right)\\ +\frac{1}{C}G\left(t\right)\left(V\left(t\right)-E\right)\\ +g\cdot \delta \cdot \exp\left(\frac{V\left(t\right)-V_{\text{T}}}{\delta }\right)-w\left(t\right)\\ \frac{d}{dt}w\left(t\right)=\frac{c}{\tau_{w}}\left(V\left(t\right)-E_{\text{L}}\right) \end{array}$$

For the biophysical meaning of the variables *V*_T_, δ, *g*, *c*, τ_ω_ and *w* see the original publication by Brette and Gerstner (2005).

Current-based neuron models with *F* ≢ 0 are unusual because models from this category are chosen primarily for their simplicity, while conductance-based neuron models are believed to describe neuronal activity in the brain more accurately, albeit at the cost of more complex differential equations.

It should be noted here that although some neuron models are not explicitly referred to or described as *current-based* or *conductance-based* models in the literature their time evolution can still be expressed by differential equations of the mathematical forms shown in equations 2 and 3.

The choice of an appropriate solver for a given equation is a non-trivial task, as it requires deep knowledge of ordinary differential equations and numerics to assess the type of differential equation and construct an appropriate numeric solver. This choice depends not only on the form of the differential equation but also on the magnitude of the occurring parameters. For example, Rotter and Diesmann (1999) demonstrated that for neuron models that can be expressed as time-invariant linear systems, the analytical solution to the evolution of the dynamics from one time step to the next can be achieved by a matrix multiplication. If applicable, this kind of solution is to be preferred, as it is both exact and computationally efficient.

However, this approach leaves two key steps up to the modeler: firstly, analyzing the dynamics to discern what category of dynamical system it is; secondly, having performed this analysis, to construct the appropriate solver, e.g., the terms of the propagator matrix for such neurons that can be solved in this way (Rotter and Diesmann, 1999) or the configuration of an implicit or explicit numeric solver for all other neuron models. As these steps can be quite challenging to many modelers, it would be of great use to have a framework capable of automatically performing this analysis and solver construction.

In section 2 we therefore first derive compact canonical representations of the equations and their parts that allow an efficient implementation on a computer system, and then show that the distinction between current- and conductance-based, linear and non-linear, stiff and non-stiff systems of differential equations is important for automatizing the construction or selection of an optimal evolution scheme.

Our reference implementation follows the mathematical observations and is described in section 3. Section 4 demonstrates our application of the framework to some commonly used models in computational neuroscience and explains the integration of the framework into the NEST Modeling Language (NESTML; Plotnikov et al., 2016). We close with a presentation of related work in section 5 and a discussion and outlook in section 6, where we summarize possible extensions and further applications of our system.

## 2. Materials and methods

As already pointed out in the previous section, systems of differential equations describing the dynamics in neuron models can be divided into *current-based* and *conductance-based* systems. Additional distinguishing properties are whether the systems are *linear* or *non-linear, stiff* or *non-stiff*. We will now describe how these properties influence the choice of an appropriate solver.

For the current-based integrate-and-fire neuron with *F* ≡ 0, we have a first order constant coefficient linear differential equation where *I* typically satisfies a homogeneous linear differential equation of some order *n* ∈ ℕ. Any such ODE or system of ODEs can be solved analytically and efficiently as we will show in section 2.1.

When evolving systems of ODEs for conductance-based linear or non-linear ODEs, it is necessary to use a numeric integration scheme. Depending on the system at hand, it is advisable to choose either an implicit or an explicit stepping function (section 2.2).

### 2.1. Solving linear constant coefficient ODEs analytically

For simplicity we will assume *E*_*L*_ in equation 2 to be zero or to be included in one of the other terms of the right hand side. As shown by Rotter and Diesmann (1999), if *V*:ℝ → ℝ satisfies the first order constant coefficient linear differential equation

(4) $$\begin{array}{l} \frac{d}{dt}V\left(t\right)=-\frac{1}{\tau }V\left(t\right)+\frac{1}{C}I\left(t\right) \end{array}$$

with initial value *V*(0) = *V*_0_, for a function *I*:ℝ^+^ → ℝ and constants *C* (the capacitance of the membrane) and τ (the membrane time constant), and if *I* satisfies

(5) $$\begin{array}{l} {\left(\frac{d}{dt}\right)}^{n}I=\overset{n-1}{\underset{i=0}{\sum }}a_{i}{\left(\frac{d}{dt}\right)}^{i}I \end{array}$$

for some *n* ∈ ℕ and a sequence (_*a*_*i*_)*i*∈ℕ_ ⊂ ℝ, an analytical solver can be constructed in the form of a propagator matrix.

Here, we show how to evaluate the dynamics to discern whether *V* and *I* do indeed satisfy the conditions stated above, and how to derive the evolution scheme for *V* accordingly. First, we verify that the first order differential equation, $\frac{d}{dt}V=r\left(V\right)$, for a right hand side *r*:ℝ × ℝ^+^ → ℝ, is indeed linear with a constant coefficient, i.e., that ${\left(\frac{d}{dV}\right)}^{2}r\left(V\right)=0$ and $\left(\frac{d}{dV}\right)r\left(V\right)\left(t\right)$ is constant. Second we methodically determine whether *I* satisfies a linear differential equation of some order *n*, i.e., we check whether

(6) $$\begin{array}{l} \frac{d}{dt}I=a_{0}I \end{array}$$

for some *a*_0_ ∈ ℝ by solving for *a*_0_. If no such *a*_0_ exists we check whether

(7) $$\begin{array}{l} {\left(\frac{d}{dt}\right)}^{2}I=a_{0}I+a_{1}\frac{d}{dt}I \end{array}$$

for some *a*_0_, *a*_1_ ∈ ℝ using the following procedure: we assume that *a*_0_, *a*_1_ exist such that (7) is satisfied. Then we have for some *t*_1_, *t*_2_ ∈ ℝ (for example *t*_1_ = 1, *t*_2_ = 2):

$$\begin{array}{l} \text{X}\left(t_{1},t_{2}\right):=\left(\begin{array}{cc} I\left(t_{1}\right) & \frac{d}{dt}I\left(t_{1}\right)\\ I\left(t_{2}\right) & \frac{d}{dt}I\left(t_{2}\right) \end{array}\right), \end{array}$$

$$\begin{array}{l} \text{X}\left(t_{1},t_{2}\right)\cdot \left(\begin{array}{c} a_{0}\\ a_{1} \end{array}\right)=\left(\begin{array}{c} {\left(\frac{d}{dt}\right)}^{2}I\left(t_{1}\right)\\ {\left(\frac{d}{dt}\right)}^{2}I\left(t_{2}\right) \end{array}\right) \end{array}$$

If det(**X**(*t*_1_, *t*_2_)) ≠ 0 we therefore know that

$$\begin{array}{l} \left(\begin{array}{c} a_{0}\\ a_{1} \end{array}\right)={\text{X}}^{-1}\left(t_{1},t_{2}\right)\cdot \left(\begin{array}{c} {\left(\frac{d}{dt}\right)}^{2}I\left(t_{1}\right)\\ {\left(\frac{d}{dt}\right)}^{2}I\left(t_{2}\right) \end{array}\right). \end{array}$$

Under the assumption that (7) is satisfied and that det(**X**(*t*_1_, *t*_2_)) ≠ 0 this gives us *a*_0_ and *a*_1_. If our second assumption is not satisfied we can easily chose *t*_1_ and *t*_2_ so that it is. We can now determine whether the first assumption is correct by inserting the calculated values for *a*_0_ and *a*_1_ and checking if the following equation is true:

(8) $$\begin{array}{l} {\left(\frac{d}{dt}\right)}^{2}I-a_{0}I-a_{1}\frac{d}{dt}I=0 \end{array}$$

Now, if such *a*_0_ and *a*_1_ exist, they are unique, as *I* and $\frac{d}{dt}I$ are linearly independent, since there was no *a*_0_ ∈ ℝ such that (6) was satisfied. If *a*_0_ and *a*_1_ do not satisfy (8), we check methodically if constants (*a*_*i*_)_*i*∈ℕ_ ⊂ ℝ exist, for which (5) is satisfied for *n* = 3, 4, … . Again we assume that *a*_0_, …, *a*_*n*_ ∈ ℝ exist such that (5) is satisfied. Then we have for $t=\left(t_{1},\ldots ,t_{n}\right)\in {\mathbb{R}}^{n}$ (for example *t*_1_ = 1, …, *t*_*n*_ = *n*):

(9) $$\begin{array}{l} \text{X}\left(t\right):=\left(\begin{array}{ccc} I\left(t_{1}\right) & \cdots & {\left(\frac{d}{dt}\right)}^{n-1}I\left(t_{1}\right)\\ \vdots & \ddots & \vdots \\ I\left(t_{n}\right) & \cdots & {\left(\frac{d}{dt}\right)}^{n-1}I\left(t_{n}\right) \end{array}\right), \end{array}$$

(10) $$\begin{array}{l} \text{X}\left(t\right)\cdot \left(\begin{array}{c} a_{0}\\ \vdots \\ a_{n-1} \end{array}\right)=\left(\begin{array}{c} {\left(\frac{d}{dt}\right)}^{n}I\left(t_{1}\right)\\ \vdots \\ {\left(\frac{d}{dt}\right)}^{n}I\left(t_{n}\right) \end{array}\right). \end{array}$$

If det(**X**(*t*)) ≠ 0 we get

(11) $$\begin{array}{l} \left(\begin{array}{c} a_{0}\\ \vdots \\ a_{n-1} \end{array}\right)={\text{X}}^{-1}\left(t\right)\cdot \left(\begin{array}{c} {\left(\frac{d}{dt}\right)}^{n}I\left(t_{1}\right)\\ \vdots \\ {\left(\frac{d}{dt}\right)}^{n}I\left(t_{n}\right) \end{array}\right). \end{array}$$

Again, if det(**X**(*t*)) = 0 we simply use another *t*, for example *t* = (*t*_1_ + 1, …, *t*_*n*_ + 1). Then we obtain the values of *a*_0_, …, *a*_*n*_ under the assumption that (5) is satisfied for order *n*. We check whether the assumption in (5) is true by symbolically evaluating whether

$$\begin{array}{l} {\left(\frac{d}{dt}\right)}^{n}I-\overset{n-1}{\underset{i=0}{\sum }}a_{i}{\left(\frac{d}{dt}\right)}^{i}I=0. \end{array}$$

If (5) is not satisfied we go on to check

$$\begin{array}{l} {\left(\frac{d}{dt}\right)}^{n+1}I=\overset{n}{\underset{i=0}{\sum }}a_{i}{\left(\frac{d}{dt}\right)}^{i}I \end{array}$$

for some *a*_0_, …, *a*_*n*+1_, and so on. This way, for every *I* that satisfies (5) for order *n* we can determine the factors *a*_0_, …, *a*_*n*_. Then we can rephrase (4) as the *homogeneous* differential equation

(12) $$\begin{array}{l} \frac{d}{dt}\text{y}\left(t\right)=\text{A}\text{y}\left(t\right) \end{array}$$

with initial values **y**(0) = **y**_0_, $\text{y}=\left(\frac{d^{n-1}}{dt^{n-1}}I,\frac{d^{n-2}}{dt^{n-2}}I,\ldots ,I,V\right)$ and

(13) $$\begin{array}{l} \text{A}=\left(\begin{array}{cccccc} a_{n-1} & a_{n-2} & \cdots & \cdots & a_{0} & 0\\ 1 & 0 & \cdots & 0 & 0 & 0\\ 0 & \ddots & \ddots & \vdots & \vdots & \vdots \\ \vdots & \ddots & \ddots & 0 & 0 & 0\\ 0 & 0 & \ddots & 1 & 0 & 0\\ 0 & 0 & \cdots & 0 & \frac{1}{C} & -\frac{1}{\tau } \end{array}\right) \end{array}$$

Thus for *n* = 1 we have

$$\begin{array}{l} \text{A}=\left(\begin{array}{cc} a_{0} & 0\\ \frac{1}{C} & -\frac{1}{\tau } \end{array}\right) \end{array}$$

and for *n* = 2 we have

$$\begin{array}{l} \text{A}=\left(\begin{array}{ccc} a_{1} & a_{0} & 0\\ 1 & 0 & 0\\ 0 & \frac{1}{C} & -\frac{1}{\tau } \end{array}\right) \end{array}$$

As it can be both more convenient and computationally more efficient when **A** is a *lower triangular* matrix we give an alternative choice of **A** and **y**, where **A** is a triangular matrix:

(14) $$\begin{array}{l} \text{A}=\left(\begin{array}{ccc} a_{1}+x & 0 & 0\\ 1 & -x & 0\\ 0 & \frac{1}{C} & -\frac{1}{\tau } \end{array}\right) \end{array}$$

where

(15) $$\begin{array}{l} x=-\frac{a_{1}}{2}+\sqrt{\frac{a_{1}^{2}}{4}+a_{0}} \end{array}$$

and

(16) $$\begin{array}{l} \text{y}=\left(\frac{d}{dt}I+xI,I,V\right). \end{array}$$

Then we can determine the solution **y** at *t* ∈ ℝ^+^ using the matrix exponential:

(17) $$\begin{array}{l} \text{y}\left(t\right)=e^{\text{A}t}{\text{y}}_{0} \end{array}$$

We can rephrase this to obtain an incremental formulation which allows the evolution of the system by a single calculation of *e*^**A***h*^ for a fixed step size *h* ∈ ℝ^+^:

$$\begin{array}{l} \text{y}\left(t+h\right)=e^{\text{A}\left(t+h\right)}\cdot {\text{y}}_{0}=e^{\text{A}h}\cdot {\text{y}}_{t}. \end{array}$$

It is important to note here that the exact integration of (2) depends on the exact calculation of *e*^**A***h*^. Let *I*(*t*) be the sum of currents elicited by all incoming spikes at all grid points for times *t*_*i*_ ≤ *t*,

$$\begin{array}{l} I\left(t\right)=\underset{i\in \mathbb{N},t_{i}\leq t}{\sum }\underset{k\in S_{t_{i}}}{\sum }I_{k}\left(t\right), \end{array}$$

where $I_{k}\left(t\right)={\hat{\iota }}_{k}\iota \left(t-t_{i}\right)$, for *t* ∈ ℝ^+^. ${\hat{\iota }}_{k}$ is the *synaptic weight* of synapse *k* and ι satisfies the differential equation 5 on ℝ^+^ for some constants (_*a*_*i*_)*i* ∈ ℕ_ ⊂ ℝ and some *n* ∈ ℕ. Then *I* satisfies the differential equation 5 on ${\mathbb{R}}^{+}\backslash \left\{t_{1},\ldots ,t_{k}\right\}$. Therefore we can consider *I* as the solution of the differential equation 5 on the intervals (0, *t*_1_), (*t*_1_, *t*_2_), …  with suitable initial values. For *t* ∈ (*t*_*i*−1_, *t*_*i*_) we can calculate

$$\begin{array}{l} \text{y}\left(t\right)=e^{\text{A}\left(t-t_{i-1}\right)}{\text{y}}_{t_{i-1}}. \end{array}$$

At time *t*_*i*_, for *i* ∈ ℕ, the differential equation 5 is not satisfied because ι does not satisfy the equation at *t* = 0, but we get *I*(*t*_*i*_) by continuous continuation to the boundary of the interval (*t, t*_*i*_). The derivatives of *I* contained in **y** must be updated by initial values of additional spikes at time *t*_*i*_, meaning for **P**(*h*) = *e*^**A***h*^

$$\begin{array}{l} \text{y}\left(t_{i}\right)=\text{P}\left(h\right)\text{y}\left(t_{i-1}\right)+{\text{x}}_{{\text{t}}_{\text{i}}}, \end{array}$$

where

$$\begin{array}{l} {\text{x}}_{{\text{t}}_{\text{i}}}=\text{T}\left(\begin{array}{c} {\left(\frac{d}{dt}\right)}^{n}\iota \left(0\right)\\ \vdots \\ \frac{d}{dt}\iota \left(0\right)\\ 0\\ 0 \end{array}\right)\underset{k\in S_{t_{i}+h}}{\sum }{\hat{\iota }}_{k}. \end{array}$$

Here **T** ∈ ℝ^*n*+1^ × ℝ^*n*+1^ is such that

$$\begin{array}{l} \text{y}=\text{T}\left(\begin{array}{c} {\left(\frac{d}{dt}\right)}^{n-1}I\\ \vdots \\ I\\ V \end{array}\right). \end{array}$$

**T** is the identity matrix when **y** is chosen as the vector of derivatives as in equations 12 and 13 but it may well be non-trivial, e.g., when **y** is chosen as in equation 16.

Now we know an analytical and efficient way to evolve any linear constant coefficient ODE containing the convolution of the solution of a linear homogeneous ODE and a weighted spike train.

#### 2.1.1. Adding a constant external input current

A common requirement in neuroscientific modeling is to add a bias current to neurons. We will now show how to solve the differential equation when we have an additional constant external input current *I*_E_:

$$\begin{array}{l} \frac{d}{dt}V\left(t\right)=-\frac{V\left(t\right)}{\tau }+\frac{1}{C}\left(I\left(t\right)+I_{\text{E}}\right),V\left(0\right)=V_{0} \end{array}$$

As shown above, we can solve

(18) $$\begin{array}{l} \frac{d}{dt}V_{1}=-\frac{V_{1}\left(t\right)}{\tau }+\frac{I\left(t\right)}{C},V_{1}\left(0\right)=V_{1_{0}}. \end{array}$$

Consider the following differential equation,

(19) $$\begin{array}{l} \frac{d}{dt}V_{2}=-\frac{V_{2}\left(t\right)}{\tau }+\frac{I_{\text{E}}}{C},V_{2}\left(0\right)=V_{2_{0}}, \end{array}$$

where τ, *C* and *I*_E_ are constants. By *variation of constants* (Walter, 2000) we have a solution of (19):

$$\begin{array}{l} V_{2}\left(t\right)=\left(\frac{I_{\text{E}}\tau }{C}e^{t/\tau }+V_{2_{0}}\right)e^{-t/\tau }\\ =\frac{I_{\text{E}}\tau }{C}+V_{2_{0}}e^{-t/\tau }, \end{array}$$

$$\begin{array}{l} V_{2}\left(t+h\right)=\frac{I_{\text{E}}\tau }{C}+V_{2_{0}}e^{-t/\tau }e^{-h/\tau }\\ =V_{2}\left(t\right)e^{-h/\tau }+\frac{I_{\text{E}}\tau }{C}\left(1-e^{-h/\tau }\right). \end{array}$$

Now we know solutions *V*_1_ and *V*_2_ of (18) and (19). Therefore *V*: = *V*_1_ + *V*_2_ solves

$$\begin{array}{l} \frac{d}{dt}V=\frac{d}{dt}\left(V_{1}+V_{2}\right)\\ =-\frac{V_{1}\left(t\right)+V_{2}\left(t\right)}{\tau }+\frac{1}{C}\left(I\left(t\right)+I_{\text{E}}\right)\\ =\frac{V\left(t\right)}{\tau }+\frac{1}{C}I\left(t\right)+\frac{I_{\text{E}}}{C}. \end{array}$$

and for **P**: **P**(*h*) = *e*^**A***h*^ the following holds

$$\begin{array}{l} V\left(t+h\right)={\text{P}}_{n+1,1}{\text{y}}_{1}\left(t\right)+\cdots \\ +{\text{P}}_{n+1,n+1}V_{1}\left(t\right)+V_{2}\left(t\right)e^{-h/\tau }\\ +\frac{I_{\text{E}}}{C}\left(1-e^{-h/\tau }\right). \end{array}$$

As the last column *a* in **A** has only one entry $a_{n+1}=\frac{-1}{\tau }$ and $\text{P}=e^{\text{A}h}=\overset{\infty }{\underset{k=0}{\sum }}\frac{{\left(\text{A}h\right)}^{k}}{k!}$,

$$\begin{array}{l} {\text{P}}_{n+1,n+1}={\left(\overset{\infty }{\underset{k=0}{\sum }}\frac{{\left(\text{A}h\right)}^{k}}{k!}\right)}_{n+1,n+1}\\ =\overset{\infty }{\underset{k=0}{\sum }}\frac{{\left(\frac{-h}{\tau }\right)}^{k}}{k!}=e^{-h/\tau }. \end{array}$$

We get:

$$\begin{array}{l} V\left(t+h\right)={\text{P}}_{n+1,1}{\text{y}}_{1}\left(t\right)+\cdots \\ +{\text{P}}_{n+1,n}{\text{y}}_{n}\left(t\right)\\ +V\left(t\right)e^{-h/\tau }+\frac{I_{\text{E}}\tau }{C}\left(1-e^{-h/\tau }\right). \end{array}$$

This method is also applicable when we have a piece-wise constant function ${\hat{y}}_{0}$ instead of a constant *I*_E_:

$$\begin{array}{l} \frac{d}{dt}V_{2}=-\frac{V_{2}\left(t\right)}{\tau }+\frac{{\hat{y}}_{0}}{C},V_{2}\left(0\right)=V_{2_{0}}. \end{array}$$

where for all *i* ∈ ℕ there is a *c*_*i*_ ∈ ℝ such that ${\hat{y}}_{0}\left(t\right)=c_{i}$ for all *t* ∈ [*t*_*i*_, *t*_*i*_ + *h*). We rephrase the problem as:

$$\begin{array}{l} \frac{d}{dt}V_{2_{i}}=-\frac{V_{2_{i}}\left(t\right)}{\tau }+\frac{c_{i}}{C},V_{2_{i}}\left(0\right)=V_{{2_{i}}_{0}} \end{array}$$

on *t* ∈ [*t*_*i*_, *t*_*i*_ + *h*) for all *i* ∈ ℕ and get

$$\begin{array}{l} V_{2}\left(t_{i}\right)=\frac{c_{i}\tau }{C}+V_{2}\left(t_{i-1}\right)e^{-h/\tau } \end{array}$$

and

$$\begin{array}{l} V\left(t_{i}\right)=V\left(t_{i-1}\right)e^{-h/\tau }+\frac{c_{i}\tau }{C}\left(1-e^{-h/\tau }\right). \end{array}$$

Now we have an exact description for how to handle the evolution of linear constant coefficient ODEs containing the convolution of the solution of a linear homogeneous ODE and a weighted spike train with an additional constant external input, that is still analytical and efficient.

#### 2.1.2. Handling sums

The approximation of postsynaptic currents observed in real brain experiments is sometimes best modeled by different functions for different synapses. We can handle the case when *I* is the sum of functions *I*_1_, *I*_2_ which satisfy a homogeneous differential equation of arbitrary order *m* and *n* in the following way. As seen above if *V*_1_ is a solution of

$$\begin{array}{l} \frac{d}{dt}V_{1}\left(t\right)=-\frac{V_{1}\left(t\right)}{\tau }+\frac{1}{C}I_{1}\left(t\right) \end{array}$$

and *V*_2_ is a solution of

$$\begin{array}{l} \frac{d}{dt}V_{2}\left(t\right)=-\frac{V_{2}\left(t\right)}{\tau }+\frac{1}{C}I_{2}\left(t\right) \end{array}$$

then *V* = *V*_1_ + *V*_2_ is a solution of

$$\begin{array}{l} \frac{d}{dt}V\left(t\right)=-\frac{V\left(t\right)}{\tau }+\frac{1}{C}\left(I_{1}\left(t\right)+I_{2}\left(t\right)\right). \end{array}$$

If, furthermore, *I*_1_ satisfies (5) for *n* ∈ ℕ

$$\begin{array}{l} V_{1}\left(t+h\right)={\text{P}}_{n+1,1}^{1}{\text{y}}_{1_{1}}\left(t\right)+\cdots \\ +{\text{P}}_{n+1,n}^{1}{\text{y}}_{1_{n}}\left(t\right)+V_{1}\left(t\right)e^{-h/\tau }. \end{array}$$

where **P**^1^ is the corresponding propagator matrix and *I*_2_ satisfies (5) for some *m* ∈ ℕ

$$\begin{array}{l} V_{2}\left(t+h\right)={\text{P}}_{m+1,1}^{2}{\text{y}}_{2_{1}}\left(t\right)+\cdots \\ +{\text{P}}_{m+1,m}^{2}{\text{y}}_{2_{m}}\left(t\right)+V_{2}\left(t\right)e^{-h/\tau } \end{array}$$

where **P**^2^ is the corresponding propagator matrix, then

$$\begin{array}{l} V\left(t+h\right)={\text{P}}_{n+1,1}^{1}{\text{y}}_{1}\left(t\right)+\cdots \\ +{\text{P}}_{n+1,n}^{1}{\text{y}}_{1}\left(t\right)\\ +{\text{P}}_{m+1,1}^{2}{\text{y}}_{2_{1}}\left(t\right)+\cdots \\ +{\text{P}}_{m+1,m}^{2}{\text{y}}_{2_{m}}\left(t\right)+V\left(t\right)e^{-h/\tau }. \end{array}$$

Therefore we just need to compute two propagator matrices to handle the sum.

### 2.2. Choice of a suitable numeric integration scheme

*Explicit methods* for solving differential equations are methods that only use already known values of the function at earlier grid points to determine the value at the next grid point. The efficiency and accuracy of explicit methods is typically sufficient for systems of ODEs used to model neuronal behavior. Popular examples of such methods are the explicit 4th order classical Runge-Kutta or the explicit embedded Runge-Kutta-Fehlberg method (Dahmen and Reusken, 2005) for the approximative solution of ODEs. Most neuron model implementations currently use explicit stepping algorithms and still achieve satisfactory results in terms of accuracy and simulation time (Morrison et al., 2007; Hanuschkin et al., 2010). However, some published models involve possibly *stiff* differential equations (e.g., Brette and Gerstner, 2005), which potentially require a different class of solvers.

Lambert (1992) defines stiffness as follows:

     If a numerical method […] applied to a system with any initial conditions, is forced to use in a certain interval of integration a steplength which is excessively small in relation to the smoothness of the exact solution in that interval, then the system is said to be stiff in that interval.

A typical case of stiffness is for example, when different parts of the solution of a system of equations decays on different time scales.

This usually comes from very different scales inherent to the ODE. These scales will reflect in the parameters of the equations, i.e., the range of constants occurring in the equations of the systems. Therefore the stiffness of a system always depends not only on the mathematical form of the equations but heavily on the magnitude of the constants occurring in them.

In principle it is possible to solve stiff equations with explicit methods, but this comes at the expense of a very small step size when using an adaptive step size algorithm and trying to achieve a certain accuracy. This in turn leads to high computational costs. For non-adaptive step size algorithms it leads to plain wrong results without the user knowing, since the algorithm still terminates, but with large error. Moreover, as the limited machine precision on a digital computer constitutes a lower bound for the step size, explicit methods usually become unstable when applied to stiff problems.

*Implicit methods*, on the other hand, do not use previous values to calculate the solution at the next grid point, but only employ them implicitly in the form of the solution of a system of equations. This makes implicit methods computationally much more costly, but usually allows a larger step size to be chosen, thus avoiding stability problems (Strehmel and Weiner, 1995).

In order to detect whether an explicit or implicit method is better suited for a given ODE we devise the following testing strategy.

First, we choose representative spike trains (drawn from a Poisson distribution) and compute approximate solutions for the given system of ODEs using an explicit and implicit method of the same order:

1. an explicit 4th order Runge-Kutta method
2. an implicit Bulirsch-Stoer method of Bader and Deuflhard (Strehmel and Weiner, 1995)

both with adaptive step size. We can then compare them with respect to the required *average step size* and *minimal step size*. In cases where the implicit method performs better than the explicit method, we have reason to believe that the ODE is stiff and that the use of an implicit method is advisable.

Although ODEs may be stiff only for very specific initial conditions, usually stiffness should be observable for a wide range of initial values, or in this case for a number of incoming spike trains (Strehmel and Weiner, 1995). By choosing many spike trains, evaluating the required step sizes for the implicit and explicit method for each of them, and comparing that to the machine precision ε, it is thus possible to detect whether the problem at hand is stiff or not. We propose the following rules for choosing an implicit algorithm:

- if the minimal step size of runs using the explicit method is close to machine precision (i.e., less than 10·ε) and this is not the case for the minimal step size of runs using the implicit method (i.e., greater than or equal to 10·ε) this is a hint that the system of ODEs is possibly stiff. In this case an explicit stepping function could become unstable or even abort, so we suggest the use of an implicit algorithm.
- if the minimal step size of runs using the explicit method is reasonably large (i.e., greater than or equal to 10·ε) we have to test two cases:
  – if the minimal step size of runs of the implicit method is very small (i.e., less than 10·ε), we suggest using an explicit method.
  – if the minimal step size of runs of the implicit method is large (i.e., greater than or equal to 10·ε), we go on to check if the average step size of runs using the implicit algorithm is much larger than the average step size of runs using the explicit algorithm. If this is the case, this again indicates that the system of ODEs is stiff and therefore choosing an implicit evolution method is advisable.

For a non-stiff system of ODEs, the computation time of an explicit algorithm should be lower, as it does not require the solution of a system of equations (Dahmen and Reusken, 2005). Therefore the choice of an explicit evolution method is sensible in cases where none of the above conditions are met. The algorithm that follows from these rules is depicted in **Figure 2**.

## 3. Reference implementation

In order to automate the process of finding the most appropriate solver for a given system of ODEs on a computer, we have designed and implemented an analysis toolbox in Python (http://github.com/nest/ode-toolbox). It builds on the formal mathematical foundations introduced in the previous sections and uses SymPy (Meurer et al., 2017) to carry out symbolic mathematical tests and transformations. To achieve a high degree of portability and re-usability, the input to the algorithm is given either in the form of JSON files or Python dictionaries, which specify equations, parameters and additional properties (for an example, see section 3.4). These two means of input allow an easy embedding of the toolkit into third-party tool chains and enable us to leverage the Python and SymPy parsers, which delegates all syntax checking and exception handling to well established and tested tools.

The algorithm expects three components in the input: (i) an ODE describing the time evolution of a state variable (e.g., *V*), (ii) a list of postsynaptic shapes (e.g., *I*) used within this ODE and specified either as functions of time or as ODEs with initial conditions and (iii) a set of parameters with default values for the equations. Fundamentally, the analysis algorithm checks the given system of ODEs for membership of the following two major categories and generates or selects an appropriate solver accordingly:

1. First order linear constant coefficient ODEs for the dynamics of a state variable (see equation 4) whose inhomogeneous part is a postsynaptic shape (i.e., satisfies equation 5) can be solved exactly using an analytical stepping scheme (section 2.1).
2. All other systems of ODEs have to be solved by a numerical solver. ODEs in this category are, for example, non-linear ODEs describing the time evolution of a state variables, or linear ODEs with an inhomogeneous part which is not a postsynaptic shape, i.e., not satisfying equation 5.

**Figure 1:**
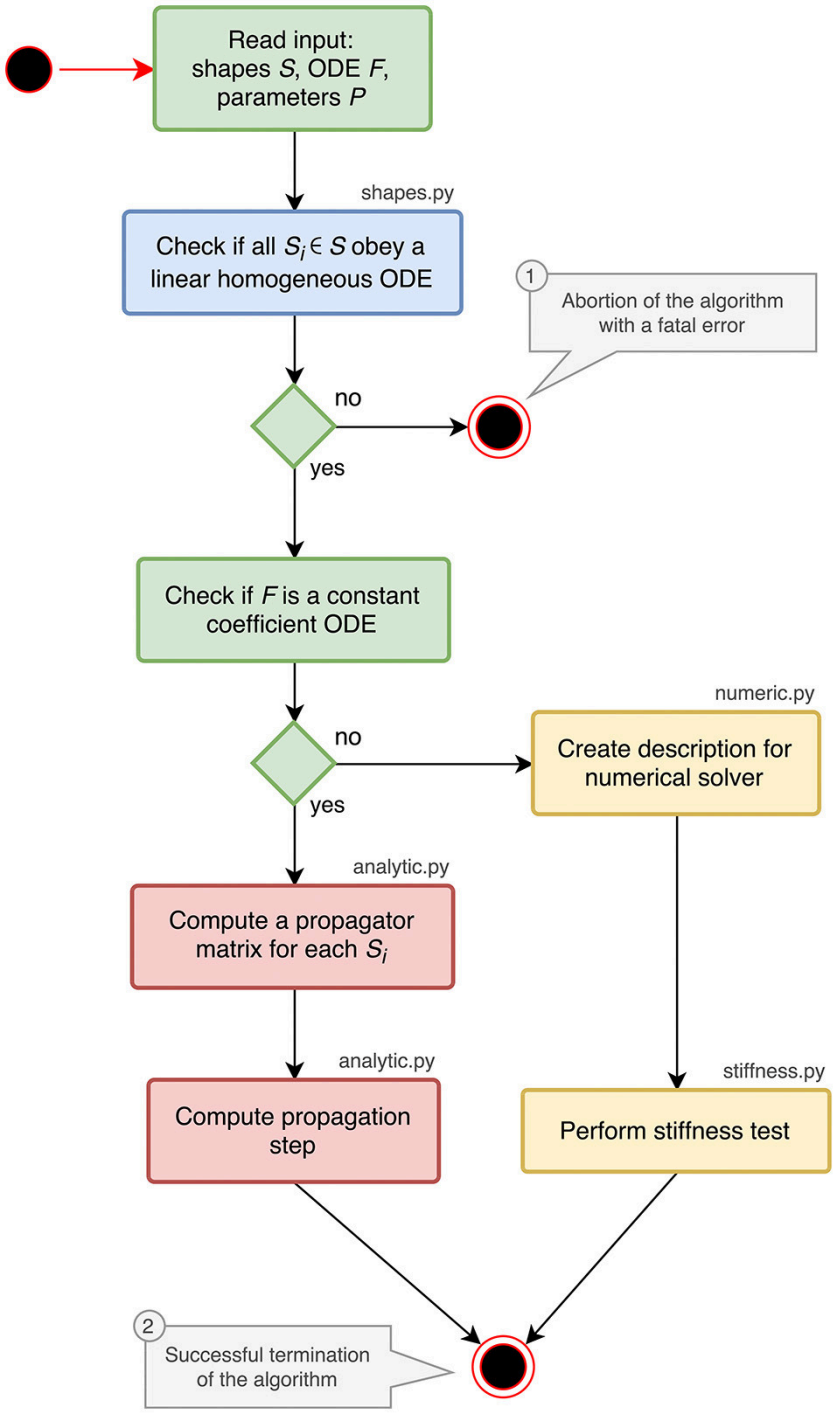
Activity diagram summarizing all steps of the ODE analysis algorithm. Steps executed in the main script of the toolbox are shown in green. The analysis of postsynaptic shapes (blue box) is detailed in section 3.1. Parts shown in red represent the generation of an analytical solver, which is described in section 3.2. The selection of a numerical stepper function is carried out by the yellow actions and explained in section 3.3.

The implementation of the analysis toolbox consists of different Python components which are introduced in the activity diagram in Figure 1. The main script orchestrates the execution of the analysis and uses the functions and classes of the different submodules:

shapes.py contains classes and functions for analyzing and storing postsynaptic shapes either given as functions of time or ODEs with initial values (blue parts in Figure 1). The main algorithm in this module is explained in section 3.1.
analytic.py provides the functionality to generate propagator matrices and compute a specification for the update step (red parts in Figure 1). A detailed description can be found in section 3.2.
numeric.py contains the code for creating a description of the update step for further processing by the stiffness tester or a numerical stepper function (upper yellow box in Figure 1).
stiffness.py implements the stiffness tester (lower yellow box in Figure 1). This module can either be used as a module within the analysis toolbox or a third-party tool, or run in a stand-alone fashion. It is explained in section 3.3 together with the preparatory steps carried out in numeric.py.

The main script starts by reading and validating the input from a JSON file or a Python dictionary. It expects the keys shapes, odes and parameters to be present in the input. For each postsynaptic shape in the shapes section, it runs the algorithm described in section 3.1, which checks if the given postsynaptic shape obeys a linear homogeneous ODE and transforms it into a canonical representation suitable for further processing. If one of the postsynaptic shapes fails the test for linearity and homogeneity, the script terminates with an error (① in Figure 1), because this class of ODEs cannot be solved easily with traditional methods as explained in section 6.

After processing the postsynaptic shapes, the script checks whether all equations in the odes section of the input are linear constant coefficient ODEs: the ODE is linear if the right hand side of the ODE differentiated twice by its symbol is zero, the coefficient of the symbol is constant if the right hand side of the ODE differentiated by its symbol is constant. If these two tests succeed, the system can be solved analytically (see section 3.2). If one of them fails, a numerical stepper has to be chosen (section 3.3). The output of the main script is again a Python dictionary or a JSON file, which contains a specification of the most appropriate solver for the given input (② in Figure 1). The remainder of this section explains the different algorithms in the submodules of the analysis toolbox.

### 3.1. Analysis of postsynaptic shapes

In the neuroscience literature, postsynaptic shapes are described either as functions of time or as ODEs with initial values. To provide users with maximum flexibility, both specifications are supported by our toolbox. Regardless of the form of the specification, each of the given postsynaptic shapes has to satisfy a linear, homogeneous ODE (equation 5) to be solved either analytically or numerically.

In case the postsynaptic shape is given as an ODE with initial values, the check for linearity and homogeneity is straightforward. For each occurring derivative of the postsynaptic shape in the shape's definition, we simply have to iteratively subtract the product of the derivative and its factor from the original definition of the postsynaptic shape and check if the final difference is zero. This check fails if the postsynaptic shape is non-linear (i.e., at least one of the derivatives occurs as a power term) or not homogeneous (i.e., not all terms of the postsynaptic shape definition are products containing a derivative of the shape). This check is implemented in the function shape_from_ode() in the shape module of the toolbox.

In case the postsynaptic shape is given as a function of time, we check whether the function obeys a linear homogeneous ODE by trying to construct such an equation together with the initial values of all relevant derivatives. This procedure is implemented in the function shape_from_function() of the shape module. We start the evaluation by checking if the postsynaptic shape function obeys a linear homogeneous ODE of order 1.


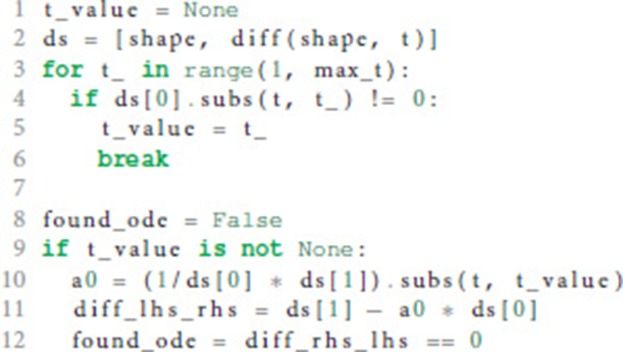


In line 10 we calculate the factor *a*_0_ from equation 6 by dividing the first derivative of the postsynaptic shape by the shape at an arbitrary point *t*. To avoid a division by zero, we have to find a *t* so that the postsynaptic shape function is not zero at this *t* (lines 3-6). Line 11 calculates the difference between the left and the right hand side of equation 6. If this difference is zero (line 12) we know that the postsynaptic shape satisfies a linear homogeneous ODE of order 1. We also know the ODE itself by calculating its initial value in line 40 below.

If the postsynaptic shape does not obey a linear homogeneous ODE of order 1, we check if the postsynaptic shape function satisfies a linear homogeneous ODE of a higher order. This test is run in a loop (line 15) that increments the order to check for each time equation 5 is not satisfied. The loop terminates if either an ODE is found or max_order iterations are exceeded. The latter check prevents expensive tests of unlikely high orders.


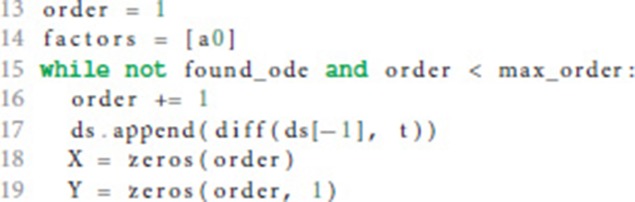


We start the loop by setting the next potential order (line 16), appending the next higher derivative of postsynaptic shape to the list of derivatives (line 17) and initializing the matrix **X** with size order × order (equation 9, line 18) and the vector **Y** with length order (right hand side of equation 10, line 19).


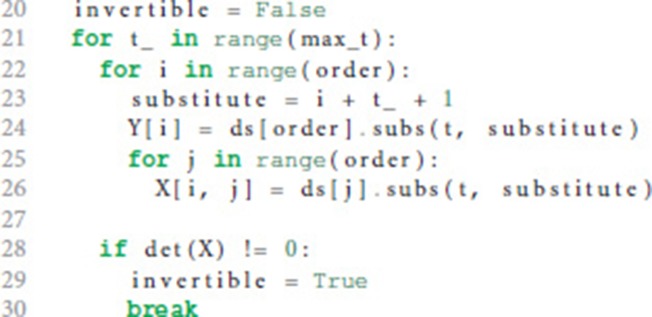


**X** and **Y** are assigned values according to equations 9 and 10 (line 24 and 26) for varying *t* = (*t*_1_, …, *t*_*n*_) (line 21) in order to find a *t* such that the matrix **X** is invertible, i.e., det(**X**) ≠ 0 (line 28). In the inner loop (lines 22-26), *t*_*i*_ is substituted so that we first try *t* = (1, …, *n*), second *t* = (2, …, *n* + 1) and so on (line 23).

If we find an invertible **X**, we calculate the potential factors *a*_*i*_ from equation 5 according to equation 11 for the current order we are checking for (factors, line 32).


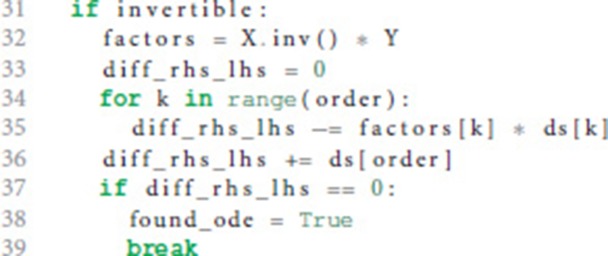


Lines 33-36 calculate the difference between the left and the right hand side of equation 5. If this difference is zero (line 37) we know that the postsynaptic shape satisfies an linear homogeneous ODE of order order.

If we do not find an ODE during the execution of the while loop, we terminate the algorithm with an error (① in Figure 1). If we do, we can go on to calculate the initial values of the postsynaptic shape equation by substituting *t* by 0 for all derivatives of the postsynaptic shape, which fully defines the found ODE.


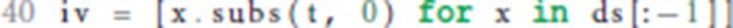


In the case of successful termination, the functions shape_from_ode() and shape_from_function() both return a Shape object to the main script of the toolbox, which encapsulates all attributes of the postsynaptic shape required for further processing.

### 3.2. Generation of an analytical evolution scheme

If the ODE describing the update of a state variable was found to be a constant coefficient ODE and all postsynaptic shapes obey linear homogeneous ODEs, we can solve the system of ODEs analytically according to section 2.1. To this end, the module analytic provides a class Propagator, which has two member functions corresponding to the two steps required for the generation of an analytical evolution scheme.

The function compute_propagator_matrices() takes an ODE and a list of Shape objects and computes a propagator matrix (equation 17) for each postsynaptic shape. These matrices can be used to evolve the system from one point to the next. The basic idea here is to populate the matrix **A** using the factors of the derivatives (factors, computed in lines 12 and 31 of the code in section 3.1), the factor of the postsynaptic shape used in the ODE for the state variable (ode_shape_factor) and the factor of the symbol of the ODE (ode_sym_factor). For the equation

$$\begin{array}{l} \frac{d}{dt}V=\frac{1}{\tau }\cdot V+\frac{1}{C_{1}}\cdot I_{1}+\frac{1}{C_{2}}\cdot I_{2} \end{array}$$

ode_sym_factor would thus be $\frac{1}{\tau }$. It is calculated using the following line of code:

1 ode_sym_factor = diff (ode_def, ode_symbol)

ode_shape_factor would be $\frac{1}{C_{1}}$ for postsynaptic shape *I*_1_ in the example equation and $\frac{1}{C_{2}}$ for *I*_2_. As these factors and other parameters depend on the postsynaptic shape, we run the following code in a loop (omitted for better readability), each iteration assigning the current Shape object to the variable shape:


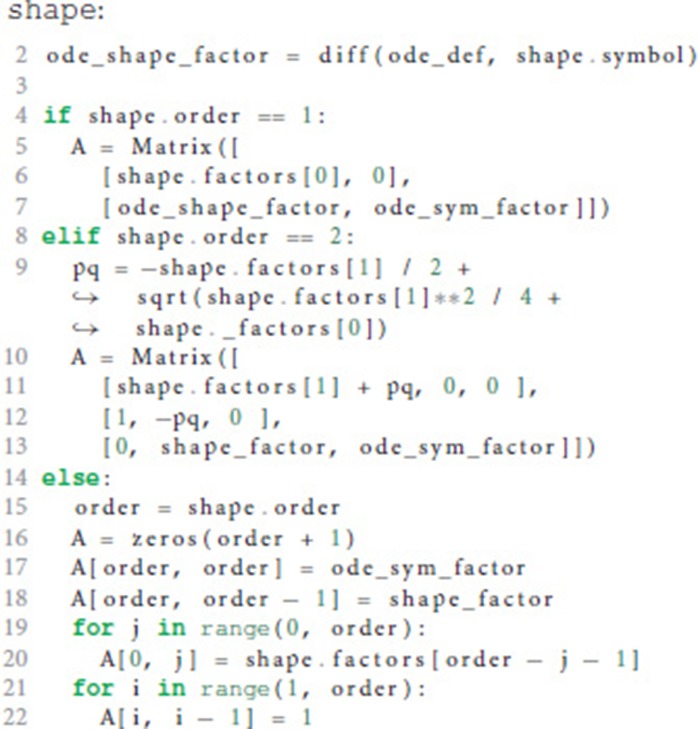


Line 2 computes the ode_shape_factor for the current postsynaptic shape. In order to make the calculation of the solution more efficient (i.e., using fewer arithmetic operations on a computer), compute_propagator_matrices() creates a lower triangular matrix for postsynaptic shapes of order 1 and 2 (lines 5-7 and 9-13, respectively) as explained in equation 14 and a generic matrix for all higher orders according to equation 13 (lines 15-22). The variable pq in line 9 corresponds to equation 15.

The propagator matrix for each postsynaptic shape can now be computed by taking the matrix exponential of the matrix **A** multiplied by the update step size *h*:

23 propagator_matrices. append (exp (A * h))

The second function of the Propagator class, compute_propagation_step(), takes the list of propagator matrices and postsynaptic shapes and computes a calculation specification that can be executed to actually perform the system update. As this function merely runs a loop over all propagator matrices and generates the update instructions as a list of strings, the code is omitted here.

### 3.3. Finding an appropriate numerical solver

In case the differential equation describing the dynamics of a state variable was not found to be a linear constant coefficient ODE, the system must be evolved using a numerical stepping scheme as explained in section 2. Instead of a full calculation specification, as produced for the analytical solution in section 3.2, the numeric module of the toolbox just passes the specification of ODEs from the input and the Shape objects created by the algorithm in section 3.1 on to the stiffness tester, which is implemented in the stiffness module.

The stiffness tester uses the standard Python modules SymPy and NumPy for symbolic and numeric calculations. For evolving the ODEs during the test procedure, it currently uses PyGSL, a Python wrapper around the GNU Scientific Library (GSL; Gough, 2009). This library was chosen over more pythonic alternatives such as SciPy due to its more comprehensive selection of ODE solvers.

**Figure 2:**
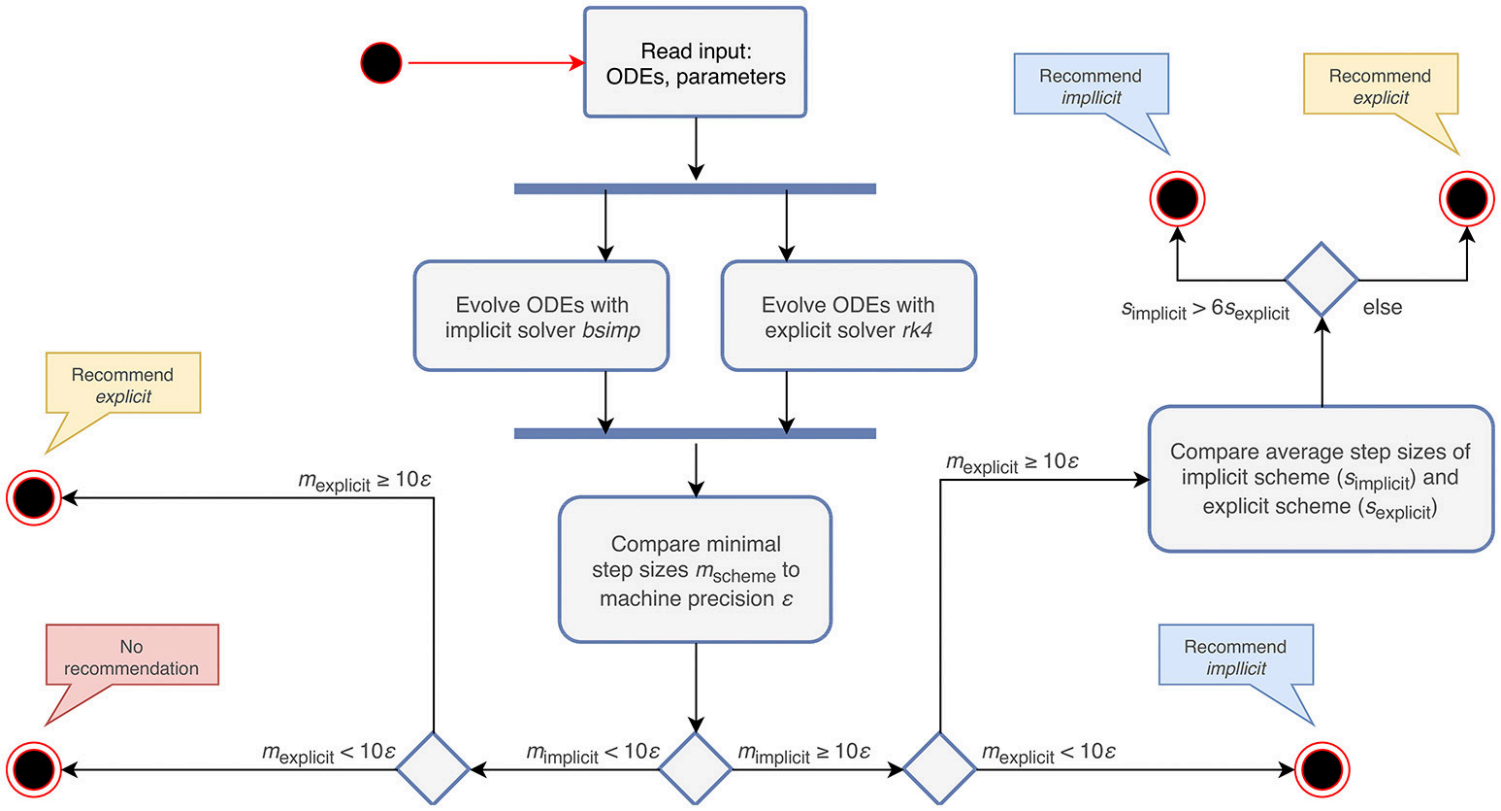
Activity diagram summarizing the steps taken to recommend an appropriate numerical stepping scheme. The input to the algorithm are the ODEs and their parameters. After evolving the system of ODEs in parallel with an implicit and an explicit solver, it compares the minimal step sizes (*m*_scheme_) of each scheme with the machine precision (ε). Depending on the outcome of the comparison, it recommends an appropriate stepping scheme (explicit or implicit) or compares the average step sizes (*s*_scheme_) of the tested schemes. In the case that both the step size of the explicit and implicit solver are close to ε, the algorithm does not give a recommendation, but terminates with a warning instead.

The stiffness tester executes the algorithm described in section 2.2 and gives a recommendation as to whether the use of an explicit or an implicit evolution scheme is appropriate. The steps performed by the algorithm are shown in Figure 2. The choice of the factor 6 for comparing average step sizes of the explicit and the implicit schemes is motivated in section 3.3.1. For the evolution of the system of ODEs, the equations receive representative spike trains drawn from a Poisson distribution with a rate of ν = 0.1 s^−1^ and inter-spike intervals distributed around $\frac{1}{\nu }$ (Connors and Gutnick, 1990).

#### 3.3.1. Comparison of average step sizes

When comparing average step sizes of the implicit and explicit method applied to a certain set of ODEs, we assume that the set of ODEs is stiff when the average step size of the implicit method is considerably larger than the average step size of the explicit method, see section 2.2, i.e., when *s*_implicit_ > β · *s*_explicit_ for some β.

To determine an appropriate factor β, we developed a testing strategy using a well known example of a set of stiff ODEs: with *a* = −100 and initial values *y*_1_(0) = *y*_2_(0) = 1,

(20) $$\begin{array}{l} \frac{dy_{1}}{dt}=ay_{1}\\ \frac{dy_{2}}{dt}=-2y_{2}+y_{1} \end{array}$$

is a typical stiff ODE system (example taken from Dahmen and Reusken, 2005). The solution $y_{1}\left(t\right)=e^{-100t}$ decays very quickly, whereas the solution $y_{2}\left(t\right)=-\frac{1}{98}e^{-100t}+\frac{99}{98}e^{-2t}$ decreases a lot more slowly, which causes the stiffness of this system.

*y*_1_ is already reduced by four decimal places at *t* = 0.1 and *y*_1_ is practically negligible for even larger *t*. Nevertheless, it plays a major role in the calculation of *y*_2_ when using an explicit integration method. Using a simple explicit Euler method and a resolution *h* for the approximation ỹ_1_ of *y*_1_, we have the following recursive specification:

$$\begin{array}{l} {ỹ}_{1}\left(t+h\right)={ỹ}_{1}\left(t\right)-100h{ỹ}_{1}\left(t\right)=\left(1-100h\right){ỹ}_{1}\left(t\right). \end{array}$$

For $h=\frac{1}{200}$ and $t=\frac{1}{10}$ we get

$$\begin{array}{l} {ỹ}_{1}\left(1/10\right)=2^{-20}<10^{-6}. \end{array}$$

For computational efficiency, we would like to choose a larger step size for *y*_2_ since the solution decays a lot slower than *y*_1_. If we therefore choose $h=\frac{1}{2}$ to integrate *y*_2_, we get

$$\begin{array}{l} {ỹ}_{1}\left(t+h\right)=-49{ỹ}_{1}\left(t\right), \end{array}$$

causing an explosive growth in the course of the calculations.

A stiff set of ODEs will always result in the average step size of an implicit method exceeding by far the average step size of a comparable explicit method. Hence the runtime of the implicit method should be less than the explicit method's runtime. However, runtime is not solely affected by the grade of stiffness, so the stiffness of a given set of ODEs is evaluated more accurately by comparing average step sizes.

To isolate stiffness from other factors, we chose equation 20 for its simplicity. This problem is clearly stiff, as described above, and the grade of stiffness relates directly to the size of the factor *a*. Therefore it can be used as a controlled stiff problem where other effects coming from the complexity of the system do not play a role.

**Figure 3:**
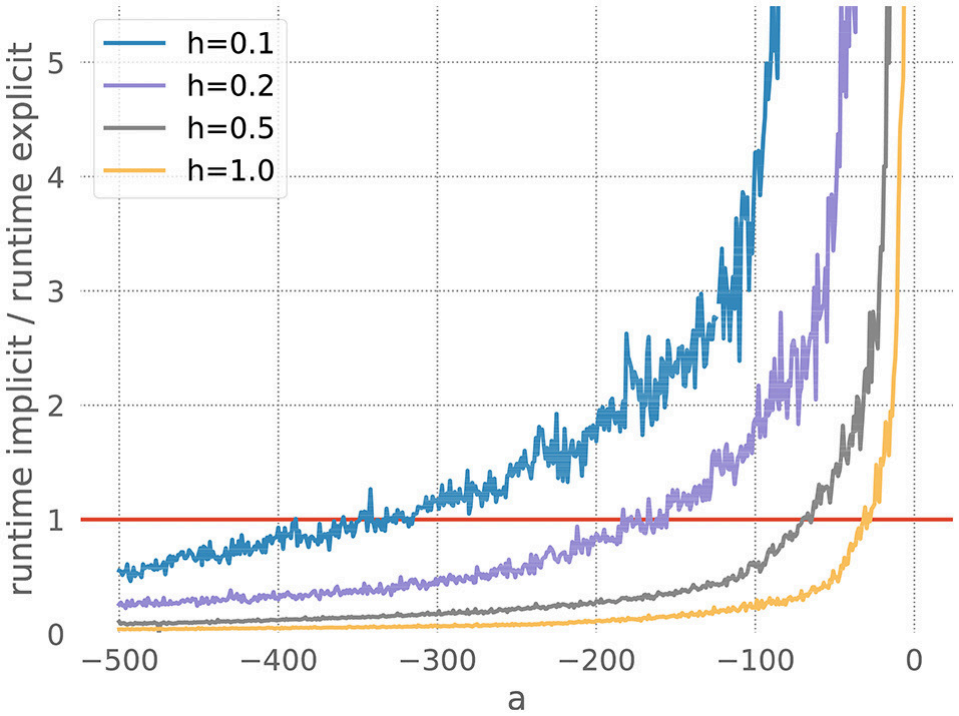
Comparison of implicit and explicit methods for a stiff ODE. Ratio of runtimes for the implicit and explicit method as a function of the factor *a* in equation 20, for varying resolutions *h* and a desired accuracy of 10^−3^. Curves averaged over 5 runs of 20 ms each. The red bar indicates when the explicit and implicit methods require the same amount of time to evolve the ODE system. Where a curve is below the red bar, the implicit method is faster than the corresponding explicit method.

We measure the runtimes of the implicit and the explicit methods (using the corresponding GSL-solvers) for five runs over 20 milliseconds each, whilst systematically varying the stiffness controlling parameters *a* and the resolution *h*. The quotient of the average implicit and explicit runtimes is shown in Figure 3.

For each measurement series, we can determine a^*^, the value of *a* for which the runtimes of the explicit and the implicit evolution scheme are the same. We then calculate the ratio of the step sizes employed by the implicit and explicit schemes at a^*^: $r^{*}=\frac{s_{\text{implicit}}\left(a^{*}\right)}{s_{\text{explicit}}\left(a^{*}\right)}$. Because in this problem the runtime, stiffness and step size are solely influenced by the factor *a*, we can consider *r* to be the borderline factor, i.e., problems with $s_{\text{implicit}}>r^{*}\cdot s_{\text{explicit}}$ are sufficiently stiff to make the implicit method faster.

For all the curves in Figure 3, we determine a value for r^*^ between 6 and 7. As some input scenarios may result in a somewhat stiffer system than that brought about by the representative spike train chosen in the stiffness tester, we choose β = 6 conservatively on the low side of the range of r^*^, to ensure that the implicit scheme is used in all stiff cases.

### 3.4. Example

The use of the toolbox as a Python module is explained in detail in the README.md file of the git repository at http://github.com/nest/ode-toolbox. Here, we demonstrate the use of the analysis toolbox by executing the script file ode_analyzer.py in a stand-alone fashion for generating a solver specification for a conductance-based integrate-and-fire neuron with alpha-shaped postsynaptic conductances. The script expects the name of a JSON file as its only command line argument:

python ode_analyzer . py iaf_cond_alpha . json

The file iaf_cond_alpha.json is shown in Listing 1. It contains the specification of one differential equation for the membrane potential V_m in the odes section in lines 3-7. This section is a list and can potentially contain multiple ODEs. The shapes section defines two postsynaptic shapes, one of which is specified as a function of time (g_in, lines 10-14), the other as an ODE with initial conditions (g_ex, lines 15-20). The parameters and their default values are given in the parameters dictionary in lines 22-33. This dictionary maps default values to parameter names and has to contain an entry for each free variable occurring in the equations given in the odes or shapes sections.

**Listing 1:**
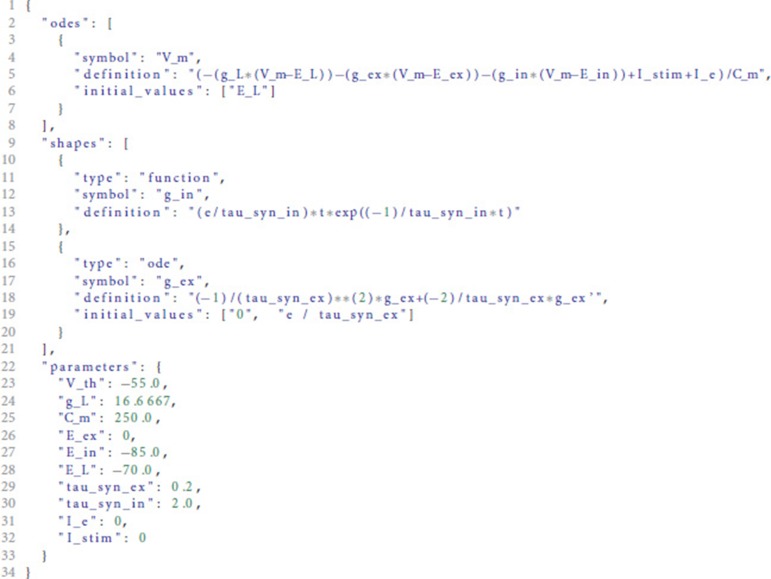
Example JSON file as input to the analysis toolbox. The file contains three entries: odes describing the ODEs of the system, shapes containing the postsynaptic shapes used in the ODEs and parameters specifying the parameters and default values for the differential equations in the shapes and odes sections.

Depending on the complexity of the ODEs and postsynaptic shapes contained in the input, the analysis may take some time. During its execution, the analysis tool prints diagnostic messages about the current processing steps. If all steps succeed, it writes the result again to a JSON file, which can be read by the next tool in the model generation pipeline to create the complete model implementation.

For the input shown in Listing 1, the analysis toolbox produces the following output:


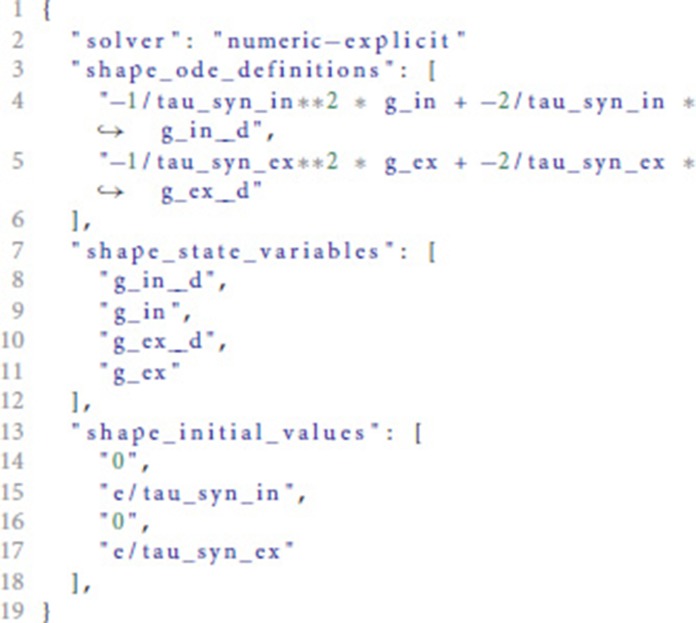


The meaning of the fields is explained in detail in the README.md of the toolbox.

## 4. Results

To evaluate the proposed framework for the semantic analysis of a system of ODEs and assessment of its stiffness we have chosen two approaches. One was to apply the stiffness tester to the neuron models currently implemented in the NEST Modeling Language (NESTML; Plotnikov et al., 2016), the other was to compare runtimes of explicit and implicit evolution schemes applied to two commonly used simplified versions of the Hodgkin-Huxley model.

The stiffness tester was integrated and successfully used in the tooling for NESTML, a domain specific language for the definition of neuron models for the neuronal simulator NEST (Gewaltig and Diesmann, 2007; Kunkel et al., 2017). NESTML is built using MontiCore (e.g., Grönniger et al., 2008; Krahn, 2010). MontiCore is a language workbench (Erdweg et al., 2013) that enables an agile and incremental implementation of lightweight DSLs including the symbol table functionality (Mir Seyed Nazari, 2017), code generation facilities (e.g., Schindler, 2012; Rumpe, 2017) and support for editors in Eclipse IDE (e.g., Krahn et al., 2007; Völkel, 2011). NEST's focus is on the simulation of the dynamics of large networks of spiking neurons (e.g., Kunkel et al., 2010; Potjans and Diesmann, 2012; van Albada et al., 2015). Neuron models in NEST are usually rather simple point neurons or models with a few electrical compartments instead of rich compartmental neurons built from morphologically detailed reconstructions. The simulator is capable of running on a large range of computer architectures ranging from laptops over standard workstations to the largest supercomputers available today (Kunkel et al., 2014).

Within NESTML, the analysis toolbox developed in sections 2 and 3 is used for the numerical analysis of neuron models defined as systems of ODEs and provides either the implementation of an efficient and accurate analytical integration scheme or recommends a good numerical solver. Therefore it allows the simulation of a large variety of biological neuron models in NEST.

As a simple yet meaningful validation of the stability checks introduced in section 2.2, we applied the stiffness tester to all neuron models currently implemented in NESTML (see https://github.com/nest/nestml/tree/master/models). The result of this evaluation is that with default parametrization, the systems of ODEs of all neuron models are non-stiff and can thus be safely integrated using an explicit numerical integration scheme without any detrimental effects on efficiency and accuracy. This is a reassuring finding, as it indicates that previous studies using these neuron models are unlikely to contain distorted results due to numeric instabilities in the integration, for a counter-example see Pauli et al. (2018).

**Figure 4:**
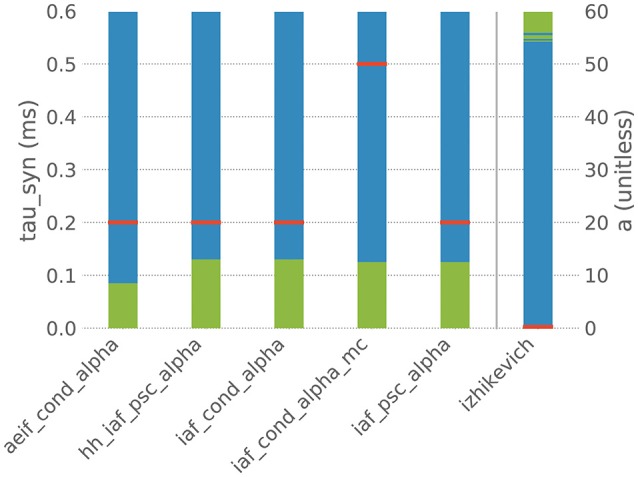
Results of the stiffness test for six neuron models from NEST. Red bars indicate the default value of the selected parameter in NEST, blue indicates the value range in which the system of ODEs evaluates as non-stiff, green indicates the range in which it evaluates as stiff. aeif_cond_alpha is a conductance-based adaptive exponential integrate-and-fire model with alpha-shaped postsynaptic conductances, hh_psc_alpha a Hodgkin-Huxley type model with alpha-shaped postsynaptic currents, iaf_cond_alpha a conductance-based integrate-and-fire neuron with alpha-shaped postsynaptic conductances, iaf_cond_alpha_mc a conductance-based integrate-and-fire neuron with alpha-shaped postsynaptic conductances and multiple compartments, iaf_psc_alpha a current-based integrate-and-fire neuron with alpha-shaped postsynaptic currents and izhikevich the model dynamics proposed by Izhikevich (2003). The test was applied to the ODE systems for varying values of the parameter tau_syn of the first five models and for the parameter a of the last model.

However, when the default parametrization is slightly altered, the stiffness test finds that some systems of ODEs are now evaluated as being stiff, which suggests that the choice of an implicit evolution scheme would be more advisable than the default choice. Figure 4 summarizes these observations for a selection of six commonly used neuron models and shows how a systematic change of one parameter in these models results in an evaluation as stiff or non-stiff.

As a second test, we apply the stiffness tester to the Fitzhugh-Nagumo and Morris-Lecar models (FitzHugh, 1961; Nagumo et al., 1962; Morris and Lecar, 1981), non-linear oscillators that include the generation of an action potential as part of the dynamics, rather than applying an artificial threshold as many point neuron models do. To assess the comparative performance of the two approaches, we vary both the stiffness controlling parameter of the model equations and the resolution *h*, as a parameter of the stiffness tester (stiffness.py; see section 3). For small values of *h*, the explicit approach is expected to exhibit a better performance, as it is relatively easy to find the solution, and the explicit approach is computationally less expensive. As *h* increases, it becomes harder to determine the correct solution, so that the more expensive, but more reliable, implicit method becomes advantageous. Alternatively, a systematic variation of the desired accuracy yields the same insight (data not shown).

**Figure 5:**
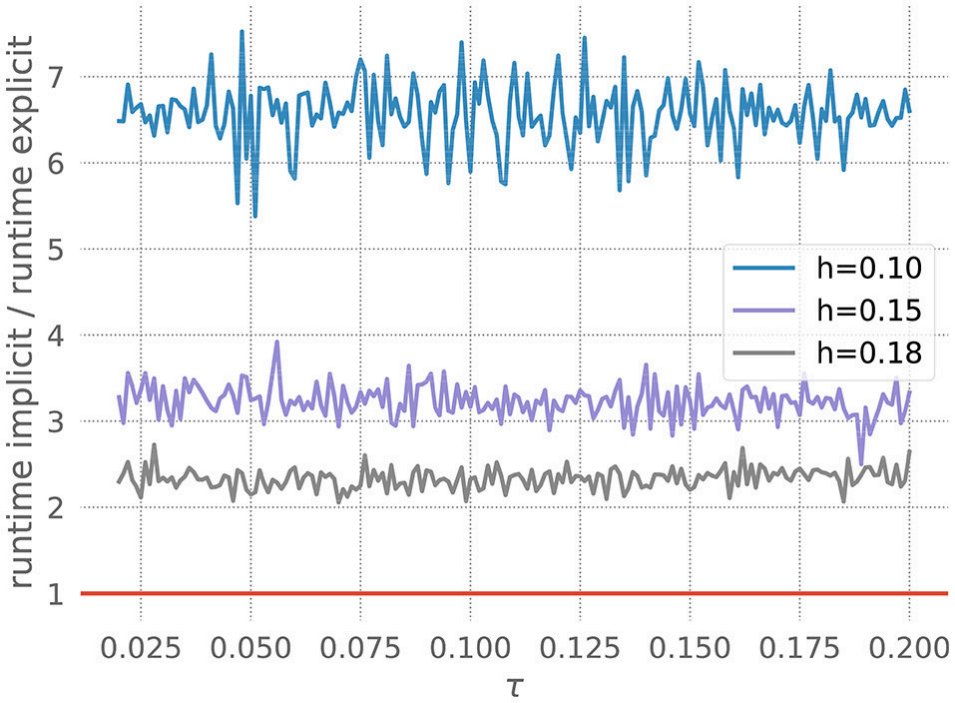
Application of the stiffness tester to the Fitzhugh-Nagumo model. Ratio of runtimes for the implicit and explicit method as a function of the factor τ in equation 21, for varying resolution *h* and a desired accuracy of 10^−5^. Curves averaged over 5 runs of 20 ms each. Red bar as in Figure 3.

Figure 5 demonstrates a comparison of the implicit and explicit methods applied to the FitzHugh-Nagumo model. The model comprises two variables, one for the membrane potential *V* and a recovery variable *W*. The dynamics are given by:

(21) $$\begin{array}{l} V^{\prime }=V-\frac{1}{3}V^{3}-W+0.25\\ W^{\prime }=\tau \left(V+0.7-0.8W\right). \end{array}$$

The figure shows the quotient of the time that the corresponding GSL-solvers for the explicit and implicit methods spent on integrating the ODE system for 20 milliseconds with a desired accuracy of 10^−5^. For all resolutions shown in Figure 5, the explicit scheme is faster, and is also the approach recommended by our toolbox. As the resolution becomes coarser (increased values of *h*), the curves shift down toward the point at which the implicit method would be faster. For *h* > 0.185, our toolbox recommends an implicit approach, and indeed in such cases the explicit scheme, as implemented by the GSL, exits with an error. This is due to the variable *V* becoming so large in one of the internal steps that it can no longer be represented by a double. For a higher required accuracy of 10^−10^, all curves shift to below the red line (data not shown), and the toolbox recommends an implicit solver for all tested resolutions.

**Figure 6:**
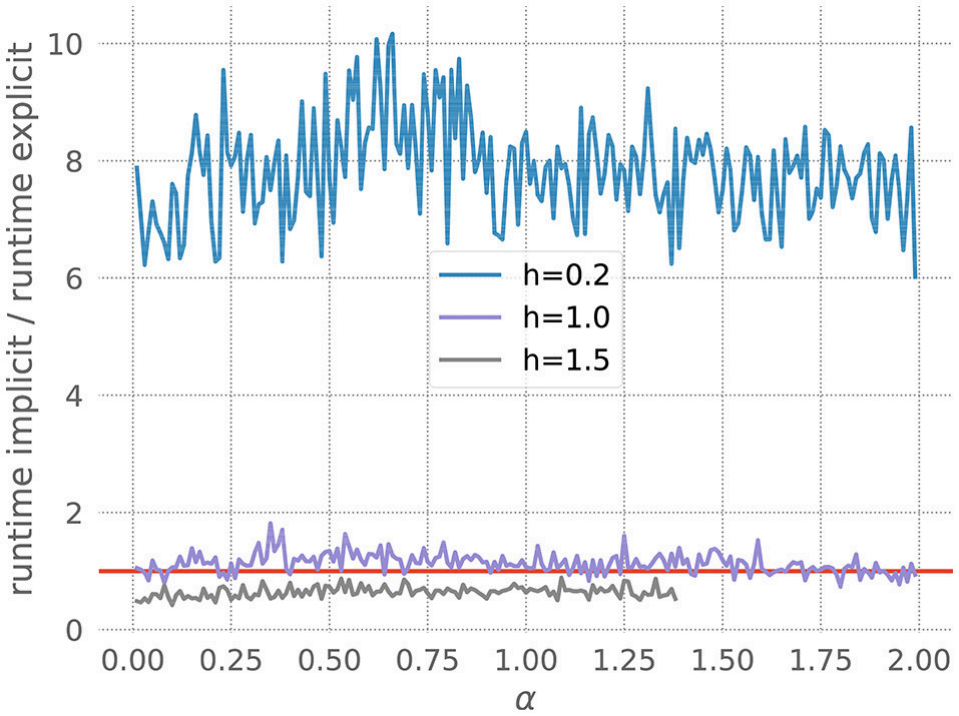
Application of the stiffness tester to the Morris-Lecar model. Ratio of runtimes for the implicit and explicit method as a function of the factor ε in equation 22, for varying resolution *h* and a desired accuracy of 10^−5^. Curves averaged over 5 runs of 20 ms each. Red bar as in Figure 3.

We apply the same approach to the Morris-Lecar model (Morris and Lecar, 1981):

(22) $$\begin{array}{l} V^{\prime }=I+2W\left(-0.7-V\right)+0.5\left(-0.5-V\right)\\ +1.1m\left(V\right)\left(1-V\right)\\ W^{\prime }=\alpha \lambda \left(V\right)\left(w\left(V\right)-W\right)\\ m\left(V\right)=\frac{1}{2}\left(1+\text{tanh}\left(\frac{V+0.01}{0.15}\right)\right)\\ w\left(V\right)=\frac{1}{2}\left(1+\text{tanh}\left(\frac{V+0.12}{0.3}\right)\right)\\ \lambda \left(V\right)=cosh\left(\frac{V-0.22}{2\cdot 0.3}\right), \end{array}$$

where *I* represents injected current. Figure 6 shows that for a resolution of *h* = 0.2, the explicit solver is faster, but for larger values of *h* the implicit solver becomes more efficient. Accordingly, our toolbox recommends explicit for the former and implicit for the latter. Note also that the explicit solver exits with an overflow error for *h* = 1.5 with values of α above 1.4. Again, the toolbox catches this risk of numerical instability and recommends the implicit scheme.

These results show that the toolbox can correctly assess where it is safe and efficient to use an explicit scheme, and where an implicit scheme would be appropriate, either for reasons of speed or for numerical stability.

## 5. Related work

In this section we compare our proposed framework for choosing evolution schemes for systems of ODEs in neural models with the corresponding approaches implemented in the simulators Brian (Goodman and Brette, 2009; Stimberg et al., 2014) and NEURON (Hines and Carnevale, 2000; Carnevale and Hines, 2006). These two simulators were chosen as they are in wide-spread use in the community. We will further consider the application of software for symbolic computation (for *exact* mathematical calculations) or scientific computing (for numerical calculations) to our setting in language modeling for neural simulators.

### 5.1. Brian

Similar to our framework, the implementation of the Brian simulator also makes a distinction between systems of ODEs that can be solved analytically and systems that can only be solved efficiently in a numeric manner. In addition to simple integrate-and-fire neurons, Brian also supports multi-compartmental neurons and neurons described by stochastic ODEs. As these types of models cannot be currently analyzed by our ODE analysis toolbox, we will not take them into account here. Instead we focus on single-compartmental deterministic neuron models as we can only draw a meaningful comparison for this group of neuron models.

In Brian, neuron dynamics can be described by a system consisting of ODEs and time-dependent functions. They are either classified as *linear*, meaning they can be solved analytically, or as *non-linear*, meaning they cannot be solved analytically and must be solved numerically using the *forward Euler method* (if not stated otherwise by the author of the model). In theory, linear constant coefficient ODEs can be solved analytically by Brian. However, if the dynamics of a neuron are described using a non-constant function of time rather than an ODE defining this function they are always solved numerically. This could be improved by using our proposed framework, which allows an analytical solver to be generated even for a system consisting of time-dependent functions that satisfy a linear homogeneous ODE and feed into a linear constant coefficient ODE. Our framework thus allows an analytical evolution for a larger class of neuron dynamics. In particular, our framework seems to be more robust with respect to the use of several different postsynaptic shapes, as they are treated seperately in contrast to Brian's approach, where the system is analyzed by SymPy as a whole.

All systems of ODEs in Brian that are not evolved by an analytical evolution scheme are by default evolved using the simple Euler method. To circumvent this, it is possible to choose a numerical evolution scheme from a list of other methods. This approach works well for users who are aware of the numerical consequences of their choice of solver but can be problematic for scientists who lack the ability to weigh up the advantages and disadvantages of different numerical evolution schemes for their particular system of ODEs. Moreover, as demonstrated in Figure 3, the choice of an appropriate evolution scheme might depend on the exact parameters for the ODEs and thus not be obvious even for an advanced user.

### 5.2. NMODL

NMODL is the model specification language of the NEURON simulator. NEURON was created for describing large multi-compartmental neuron models and thus also supports a wider range of models than our proposed framework currently does. We will again only contrast those types of models for which a comparison is meaningful.

For linear systems of ODEs, NMODL chooses an evolution method that propagates the system by evolving each variable under the assumption that all other variables are constant during one time step. In many cases this approach approximates the true solution well, but it is still less accurate than an actual analytical solution. For all other systems of ODEs, i.e., all non-linear ODEs, an implicit method is chosen, regardless of the exact properties of the equations to guarantee an evolution of stiff ODEs without causing numeric instabilities. This is a robust solution but may lead to excessively large simulation run times in cases where the choice of an explicit evolution scheme for non-stiff ODE systems would be sufficient.

### 5.3. Software for symbolic computation and scientific computing

There are a number of high quality and widely used applications available for symbolic computation, most notably *Wolfram Mathematica* (Benker, 2016), *Modelica* (Tiller, 2001), and *Maple* (Westermann, 2010). All three provide frameworks for solving ordinary differential equations both symbolically and numerically. Here, we will briefly describe their capabilities and limitations for both symbolic and numeric integration of systems of ODEs.

#### 5.3.1. Symbolic integrators

At first appearance the integration schemes provided by the programming languages (or in the case of Modelica, modeling language) seem appropriate for the task addressed in our study. As discussed in section 1, the ordinary differential equations used to define neuron models and to describe their dynamical behavior are typically linear (though not homogeneous and not linear with a constant coefficient) and can in several cases be solved analytically by any of the programs above. However, for the specific requirements related to neural simulations, there are several reasons why they are not entirely well suited.

Firstly, neurons receive input that generally changes in every integration step due to the arrival of incoming spikes, thus changing the differential equations to be solved. Although each of these differential equations can be integrated easily using, e.g., Wolfram Mathematica, none of these frameworks provide a general, exact solution for each integration step, that takes a run-time generated varying input into account. The next two points are related to the size of neural systems commonly investigated. Spiking neuronal network models often contain of the order of 10^3^–10^5^ neurons, and sometimes substantially more (Kunkel et al., 2014). Calling external software for symbolic computation of ordinary differential equations during run time for each neuron is therefore often too costly. Moreover, for large models, the simulation software is likely to be deployed on a large cluster or supercomputer. The aforementioned applications are typically not installed on such architectures, whereas Python is a standard installation, providing the package SymPy, which is sufficient for symbolic computation in this context.

#### 5.3.2. Numerical integrators

There are a number of approaches to automatically select numeric integrators depending on whether the problem is stiff or non-stiff (Petzold, 1983; Shampine, 1983, 1991). These approaches are typically designed to switch integration schemes during runtime when the problem changes its properties. All of them rely in one way or another on the behavior of the Jacobian matrix evaluated at the point of integration. Typically, the methods try to approximate the dominant eigenvalue of the Jacobian with a low cost compared to that of the stepping algorithm. However, for a spiking neural network simulation, the determination of the stiffness of the system, and thus the solver, should occur before the simulation starts, as to minimize runtime costs.

Thus the question remains whether it would be possible to carry out these kind of tests during generation of the neuron model. Applying the test to a large number of randomly selected values of the state variables, or carrying out a number of test runs using representative spike trains would allow to work around the fact that the solution up to a given point is not yet known. However, as these tests rely on determining the stiffness through the properties of the Jacobian, they would still not be completely precise. As we have the advantage of effectively no computational constraints during generation of the neuron model, there is thus no advantage by using such a low-cost strategy. In our approach we compute the solution using both explicit and implicit schemes and compare their behaviors a posteriori, thus obtaining an accurate assessment of the appropriate solver for a given set of parameters.

In addition, as for symbolic integration, the packages that provide such stiffness testing capability for numeric integration do not provide a framework for handling a run-time determined variable input due to incoming spikes. Thus we conclude that the specific problem addressed by our toolbox lies outside the scope of general purpose symbolic and numeric integration packages.

## 6. Discussion

We have presented a novel simulator-independent framework for the analysis of systems of ODEs in the context of neuronal modeling and provided a reference implementation for the selection and generation of appropriate integration schemes as open source software.

In this section we will summarize the restrictions of our framework, discuss alternative ideas for the implementation and describe possible future additions.

The framework we propose is currently limited to the analysis of equations for non-stochastic single-compartmental integrate-and-fire neuron models. The reason for this is that the analysis toolbox was developed in the context of the NESTML project, in which we put our main focus on the class of neurons presently available in the NEST simulator. The extension of the framework to other classes of neurons is one of our current research objectives. In particular, this work includes support for systems of stochastic ODEs. The symbolic analysis of neuron ODEs enables generation of the sophisticated C++ neuron implementation that switches between implicit and explicit solvers at run-time of the neurons depending on the runtime performance of the particular solver. This functionality will be integrated in upcoming releases of NESTML.

Another restriction of the framework is that it can only analyze systems of ODEs with postsynaptic shapes that obey a linear homogeneous ODE. This is due to the fact that evolving a system including postsynaptic shapes as functions of time rather than functions defined as ODEs would result in a very long sum of multiple linear combinations of shifts of this function for each incoming spike. Evaluating such a sum would make the evolution of the system containing it computationally very costly. Finding a more efficient solution for this problem is of high priority in our current work.

As noted in section 2, the calculation of *e*^*Ah*^ may become difficult to compute analytically rather than numerically if the matrix *A* becomes very large. In this case, i.e., when *e*^*Ah*^ is computed as a numerical approximation, the integration scheme is, strictly speaking, not analytical. Here it might be sensible to look into other numerical methods, e.g., integrating the system of ODEs using a quadrature formula of order 5 and thereby obtaining an accuracy of 10^−8^ despite the use of a numerical scheme.

When comparing implicit and explicit integration schemes, we compare the *average step size* and the *minimal step size* of the respective schemes. An alternative possibility would be to use fixed step sizes instead and compare the results of the explicit and implicit schemes using the results of the implicit scheme as a reference. This could be implemented alongside our current stiffness tester to provide a higher degree of certainty.

As pointed out in section 4, the stiffness of a system of ODEs depends greatly on its parametrization. Therefore it might be a useful extension to run the stiffness test not only during the generation of the model code, but also when instantiating the model in a simulator, and when model parameters are changed. This would, however, require a call to the analysis toolbox at run time, which might not be easily possible on all machines a particular simulator may run on. For example, in a supercomputer environment, job allocations are usually fixed, and not all libraries required by the toolbox may be available. An alternative solution to the problem could be to run the stiffness test for varying parameters during the generation phase of the model. This way the analysis toolbox could create a lookup table, mapping parameter values to the most appropriate integration scheme.

Another possible extension of the current framework could be to implement implicit and explicit integration schemes for evolving the systems of ODEs during the stiffness analysis, and thereby gain independence of PyGSL, which can be challenging to install. These custom implementations could be tailored to our specific requirements and give us more control over the integration scheme and the exact methodology for adaptive step size control.

The current implementation of the framework only supports fixed thresholds for the detection of spikes and evaluates the spiking criterion on a fixed temporal grid. A part of our current work is to evaluate more realistic scenarios, such as adaptive thresholds or precise detection of spike times in between the grid points. For a general discussion on the topic, see Hanuschkin et al. (2010).

Our presented framework is re-usable independently of NESTML and NEST. The source code is available under the terms of the GNU General Public License version 2 or later on GitHub at https://github.com/nest/ode-toolbox/ and we hope that the code can serve both as a useful tool for neuroscientists today, and as a basis for a future community effort in developing a simulator-independent system for the analysis of neuronal model equations.

## Author contributions

IB developed the mathematical derivations of the solver selection system and devised the algorithms. The reference implementation was conceived and created by IB and DP. DP integrated the framework into the NESTML system. JE and AM supervised and guided the work. The article was written jointly by all authors.

### Conflict of interest statement

The authors declare that the research was conducted in the absence of any commercial or financial relationships that could be construed as a potential conflict of interest.
